# Link between Excessive Smartphone Use and Sleeping Disorders and Depression among South Korean University Students

**DOI:** 10.3390/healthcare9091213

**Published:** 2021-09-14

**Authors:** Maidul Islam

**Affiliations:** Department of E-Trade, College of Social Science, Keimyung University, Dalseo-gu, Daegu 42601, Korea; maidul@kmu.ac.kr; Tel.: +82-53-580-5967

**Keywords:** smartphone, depression, sleeping disorder, Korean students

## Abstract

The purpose of this study was to explore the link between smartphone use and sleeping disorders and depression among university students in South Korea. South Korea has the highest mobile phone penetration rate as well as the highest rate of suicide of any of the Organization for Economic Cooperation and Development (OECD) nations, thus making this study of great importance. The core aim was to see whether the excessive use of smartphones has an association with sleeping disorders and depression. A cross-sectional analysis was performed to establish if there was any link between smartphone use and sleeping disorders and depression. Samples from 188 participants were used for this study. Data were collected using two well-established questionnaires, the Center for Epidemiologic Studies-Depression (CES-D) and the Athene Insomnia Scale (AIS), as well as a few questions on smartphone use. A few demographic questions were added to the questionnaire. The results of this study concluded that a significant relationship exists between smartphone use and depression. However, the finding of this research could not uncover a significant relationship between smartphone use and sleeping disorders among university students in South Korea. The excessive use of smartphones shows a relationship to an unhealthy lifestyle. There is a clear indication that the overuse of smartphones could be linked to depression. Furthermore, the study found that students with depression also tend to have sleeping disorders.

## 1. Introduction

**Figure 1:**
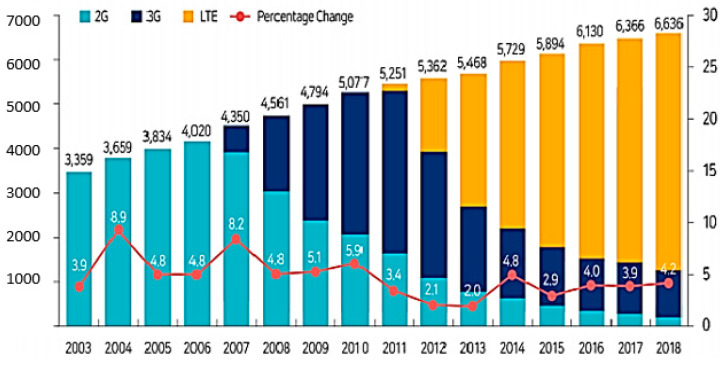
Mobile communication subscribers (source: Ministry of Science and ICT, 2019, S. Korea) (Unit: 10,000%).

**Figure 2:**
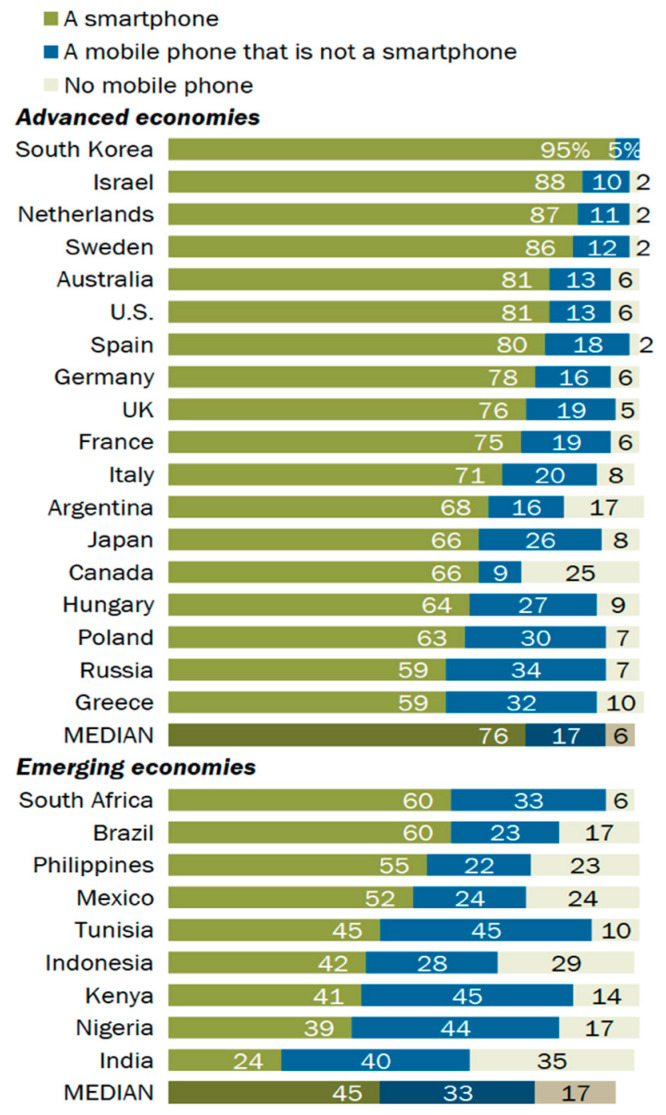
Ownership (% of adults owning) of a smartphone in advanced and emerging economies (source: PEW research, spring 2018).

**Figure 3:**
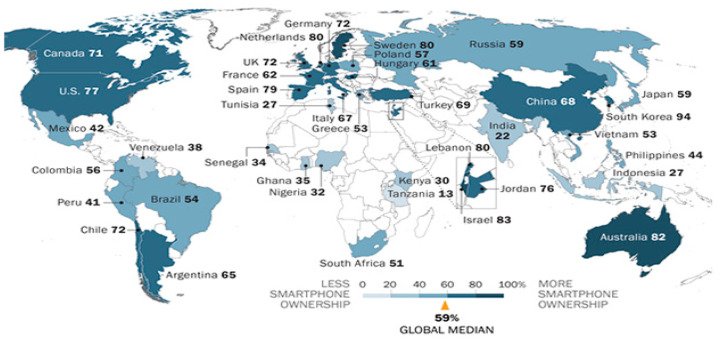
Adults who report owning a smartphone around the globe (source: Spring 2017 Global Attitudes Survey, Q65. US data from a Pew Research Center survey conducted 3–10 January 2018, China data from 2016 Global Attitudes Survey).

The number of smartphone users around the globe exceeded 3 billion in the past few years and today, it has been estimated to have reached more than 5 billion mobile phone users [1]. In addition, it has been predicted that this number will increase further in the coming few years. Several researchers also found that the young population is more attracted to the latest technology than the older one. Moreover, the smartphone has a majority of the attributes that attract the young generation [2]. Long et al. [3] stated that students considered the smartphone as a necessary good in their everyday life. It was estimated that the number of smartphone users in South Korea was 50.1 million in the year 2021 (up to March), which is up from 43.54 million in September 2015 [4]. South Koreans love using smartphones, and smartphone overdependence has increased dramatically in the last few years as a result of the launch of the 5G network in 2019. Therefore, there is no sign of the use of smartphones to decrease in South Korea. The distribution of smartphones is rapidly growing around the world and South Korea is no exception (Figure 1 and Figure 2) [5]. South Korea has the highest rate of ownership, which was 88% in 2015, increasing to 94% in 2017 [6] (Figure 3), and 98% in 2019 [7]. As per the Korea Communication Commission 2018 [8], teens and people in their twenties spend an average of between 2.21 and 2.36 h consecutively on their smartphone, which is much higher than the older adults. A recent survey conducted between July and September 2020 in South Korea shows the that age group between 13 and 19 years spends more than 4 h daily on their smartphone [9]. Therefore, it is a clear indication that the youth are engaging with the smartphone for a significant time of their day. The smartphone usage time for both genders has been rising continuously: From 1 h in 2011 to 2 h in 2014. This consistent increase in smartphone use is due to the convergence of various media, such as the live streaming of news or movies, the internet, mobile applications, location-based services, banking, online shopping, trading, and social networking, etc. Smartphones have become a very integral part of our daily life. In fact, they have created their own culture [10].

Despite its undeniable benefits, mobile phone use has been linked to a variety of risky or even worrisome behaviors. A study done by Billieux et al. [11] and Funston et al. [12] more than a decade ago suggested that one of the most serious worries of mobile phone use is that it can grow out of hand or lead to excessive use, causing problems in daily life. Financial difficulties are among the most typical negative consequences of excessive mobile phone use. Another psychological trait linked to problematic mobile phone use is impulsivity [11,13]. The Urgency, Premeditation, Perseverance, Sensation seeking (UPPS) model proposed by Whiteside and Lynam [14] has explored the connection between impulsivity and problematic mobile phone use. These dimensions demonstrate the tendency to act quickly, propensity to take into consideration an act before engaging in it, and tendency to remain engaged on a boring and difficult task. Furthermore, several other studies explained that three of the impulsive dimensions (urgency, lack of premeditation, and lack of perseverance) are closely related to the mechanism of having the ability of self-control [15,16,17]. Therefore, a low ability of self-control or the impulsive use of a smartphone potentially leads to mobile phone addiction.

Nowadays, students are using smartphones for study purposes due to the multitasking facilities and faster internet speed, which has become an acceptable phenomenon. Students are allowed to use smartphones in class despite the growing concern regarding the harm they cause [18]. In addition to these phenomena, the recent COVID-19 pandemic has forced universities to switch to online learning.

Italian professionals are using several online platforms to deliver psychological support during COVID-19 [19]. Video conferencing platforms such as Zoom have become popular tools in South Korea. Students are using smart devices, such as smartphones, to attend their classes more than ever before. Furthermore, to prevent the spread of COVID-19, many countries around the world have imposed “social distancing” rules [20]. In South Korea, a rule of not allowing private gatherings of more than five people, was imposed. A similar rule was also imposed by other countries such as Japan, Taiwan, England [21], etc. Due to this strict social distancing rule, people had no choice but to use a smartphone to keep and maintain a social connection, which can lead to the excessive use of a smartphone.

In addition to education, several previous studies have demonstrated that numerous mobile applications are very helpful tools for health promotion. These applications can track our day-to-day physical activity, provide weight control, a heartbeat check, and improve lifestyle [22,23,24]. Cho [25] have asserted that the use of communication applications on smartphones can lower the level of loneliness. However, several other studies showed a growing concern regarding the excessive use of smartphones, more specifically among the young population [26]. Academic studies done by numerous researchers have explored the relationship between a teenager’s smartphone use and their gender, health issues such as depression or anxiety, as well as other psychopathological states [27,28,29,30].

In addition to the psychopathological issues, the excessive use of smartphones causes a low academic performance and poor life satisfaction [31,32]. As per the American Psychiatric Association [32] “depression is a common and serious medical illness that negatively affects how you feel, the way you think, and how you act”. Furthermore, it was mentioned that depression causes feelings of sadness and/or a loss of interest in activities you once enjoyed.

A recent study found that due to COVID-19, people in South Korea drank less alcohol. However, they spent more time on smartphones. About 24% of the respondents spent more time playing online games after COVID-19 causing a mobile addiction [33]. According to Chiu, C.T. et al. [34] and Zarghami, M. et al. [35], the overuse of smartphones could lead to physical and psychological health problems [36,37,38]. Wang and Zhang [39] stated a growing concern of smartphone addiction among university students. A similar concern was asserted by Shoukat [40]. According to the study done by Elhai et al. [41], as well as Yu and Sussman [42], the excessive use of smartphones harms the mental health of the young generation. 

Aside from teens, adults also tend to use mobile phones at bedtime, which shows a significant association with sleeping disorders [43,44]. There are different types of sleeping disorders, such as Sleep apnea where an individual experiences abnormal patterns in breathing at the time of sleeping. Another type is Restless Legs Syndrome (RLS), which is characterized by an unpleasant sensation and a strong desire to move the legs during sleeping. However, this paper addresses a sleeping disorder as having trouble falling asleep or staying asleep all night, which is also sometimes called insomnia [45]. The excessive use of smartphones can also cause smartphone addiction, which may show a relationship to sleeping disorders [46,47,48,49,50]. Research suggests that young adults are predominantly prone to smartphone overuse [26,51], since their ability to self-regulate their use is very low, which may cause poor academic performance and lead to depression [51,52]. Previous researchers have explored that the excessive use of smartphones causes psychological issues such as anxiety among young adults [53,54]. Cain and Gradisar, [55] as well as Sohn et al. [56], found a clear association between the smartphone’s backlight and poor sleep quality. Moreover, several papers have explored the relationship between mobile phone use and depression in different countries such as China and Western countries [57,58]. Excessive screen time among European adolescents also shows an association with sleeping difficulties [59]. However, very little research has been done that focuses on Korean students specifically. Therefore, it is important to consider here the Korean case as Korea has the world’s highest smartphone penetration rate, while at a same time, Korea has the highest rate of suicide among the Organization for Economic Cooperation and Development (OECD) nations [60].

Previous studies have clearly noted the significant relationship between depression and suicide [61,62]. In addition to depression, a significant relationship has also been demonstrated between poor sleep quality and suicide [63,64,65]. However, to avoid any confusion, this paper does not try to find the relationship between smartphone addiction and suicide, but seeks to put an emphasis on the fact that depression is an important issue we all need to pay extra attention to, especially in a country where suicide as well as mobile penetration is at the extreme end among OECD nations.

Therefore, it makes a lot of sense to look into the relationship between smartphone use and sleeping disorders and depression among South Korean students. Furthermore, this paper looks into the amount of time students spend on Social Networking Services (SNS) or KakaoTalk chat through smartphones to communicate with their family and friends. In summary, this study investigates a slightly different perspective than earlier papers.

## 2. Materials and Methods

### 2.1. Participants and Procedure

An online survey was conducted to collect samples for this research paper. The respondents were randomly assigned from two Korean Universities (Keimyung University and Suwon University) in South Korea. The university of Suwon was selected since it was in Gyeonggi province, which is the most populous province in South Korea and also close to the capital city of South Korea. In addition, Gyeonggi province was located in the northern part of Korea. Whereas, Keimyung University was located in the southern part of Korea. Considering two universities from the northern and southern part of Korea may better represent the student population than simply taking two universities from one province. Therefore, these two universities were selected. Keimyung has a student strength of 24,000 and students from different parts of Daegu, Busan, and surrounding areas come to study here since it is one of the top universities in the Daegu area [66]. A cross-sectional study was performed using a self-reported questionnaire. The samples were collected mostly from the department of Humanities, Business Administration, Engineering, Medicine, and Social Science. A total of 235 questionnaires were distributed online through the university portal systems. COVID-19 was one of the critical reasons the authors could not manage to collect a sample in person. Therefore, the sample size was comparatively low. In general, it takes 5–10 min to fill out the questionnaire. Two hundred and one respondents filled out the questionnaire, with a response rate of 85.5%. There were 13 incomplete questionnaires, which were eliminated from further consideration. Data were collected and sorted very carefully. The size of the sample was comparatively small. However, in this case, a small high-quality set of respondents can also produce better conclusions than a low-quality large sample [67]. 

### 2.2. Materials

The questions regarding smartphone use, lifestyle, personal data, social support, sleeping disorders, and depression were asked in a self-administrated questionnaire. In the initial stage of the questionnaire, the participants were informed that participation in this study was entirely voluntary, and the collected data would be only used for the stated purpose. For general information, students were asked to report their gender and the number of university years. For lifestyle, the students were asked about their club activities, eating breakfast, communicating with family, wake up time, bedtime, and sleeping hours. Participants were asked to choose answers regarding taking part in specific club activities (cultural; sports; other; and none). Likewise, participants were asked about having breakfast (eat every day; sometimes do not eat) and communicating with family (everyday; sometimes do not communicate). In addition, they were asked when they wake up (before 6 a.m.; between 6 and 7 a.m.; and after 7 a.m.), when they go to bed (before 11 p.m.; between 11 p.m. and midnight; between midnight and 1 a.m.; and after 1 a.m.), and the total hours of sleep (less than 5 h; between 5 h and 6 h; between 6 h and 7 h; more than 7 h).

Regarding smartphone use, students were asked the following information: “Do you own a smartphone?” (own a smartphone; own another type of phone; none). Information regarding the time of daily smartphone usage were evaluated through the following question: “How much time do you spend in a day using your smartphone?” (0; less than an hour; 1 to 2 h; 2 to 3 h; 3 to 4 h; 4 to 5 h; more than 5 h). The answers regarding types of usage, such as e-mail, social media (Instagram, Facebook, Twitter, etc.), online chatting applications (Line, Kakao Talk, Facebook Messenger, etc.), internet searching, playing games, and watching videos were as follows: 0; less than 30 min; between 30 and 60 min; between 60 and 120 min; more than 120 min.

To quantify their depression level, participants were given a slightly modified version of the Center for Epidemiological Studies-Depression (CES-D) scale, which has 20 questions (Appendix A) with no reversed item question. The CES-D was designed by Radloff [68]. The CES-D scale is widely used internationally to check depressive symptoms in large populations [69]. Its validity and reliability were explained in an earlier paper by Shima and Kitamura [70]. This paper used the following measurement scale: 0: Rarely or never; 1: Sometimes or on rare occasions; 2: Occasionally or a moderate amount of time; 3: Most or all of the time. The points range from 0 to 33, where a greater point means more intense depression. A point of 8 or more was used to define clinically meaningful depressive symptoms [68]. Therefore, in this study, the cut-off value of 8 points was used to check students with depression. In the case of sleeping disorders, participants were asked to fill out a slightly modified version of the Athens Insomnia Scale (AIS) (Appendix B). This is a widely used scale to evaluate insomnia or, in other words, sleeping disorders [69,71]. The Cronbach’s alpha in this part of the current study was 0.75. In this scale, there were eight sections: Difficulty falling asleep; occasionally waking up during the night; waking up earlier than expected in the morning; total amount of sleeping time; overall quality of sleep; problems with sense of well-being; problems with mental and physical functioning; and sleepiness during the day. Each section was assessed as follows: 0 (no trouble) to 3 (serious trouble), and the range of total points was from 0 to 24. An AIS rating of 6 points was the prime cut-off basis for the ICD-10 diagnosis of insomnia [72]. Therefore, for this study, 6 points were defined as the cut-off point for a sleeping disorder, i.e., lower than 6 was not considered as having a sleeping disorder.

To evaluate social support, a modified version of the Multidimensional Scale of Perceived Social Support (MSPSS) was adopted, which is a seven-section self-administered questionnaire [73]. This scale measured social support from family, relatives, and friends. Every section was rated on a 7-point Likert scale, and the total was calculated by averaging the points from every section. The points ranged from 1 to 7, and the higher the number, the better the social support.

### 2.3. Statistical Analysis

The Mantel–Haenszel test was employed to analyze trends, and the Kruskal–Wallis test was employed to check the difference between smartphone use and sleeping disorders and depression. The association between smartphone use and sleeping disorders and depression was examined using Crosstabs and the correlation test. The dependent variable was sleeping disorder (0 = no problem [AIS score < 6] and 1 = sleeping disorder [AIS score ≥ 6]) or depression (0 = no problem [CES-D score < 8] and 1 = depression [CES-D score ≥ 8]). A significant rate (*p*-value) was worked out from the correlation test, input gender, university year, and factors associated with the dependent variables. In the correlation test, the different hours of smartphone use per day and hours spent on E-mail, SNS, online chats, internet, playing games, and viewing videos were included as predictors. *p*-Values < 0.05 were considered statistically significant. All statistical analyses were performed using IBM SPSS 25.0.

## 3. Results

As shown in Table 1, out of 188 students, 95 (50.5%) were male and 93 (49.4%) were female, with the school year ranging from 1 to 4. Smartphones were possessed by 99.5% of participants (*n* = 187), and 0.5% (*n* = 1) did not have smartphones. Regarding the smartphone daily use time (Table 2), around 50% (*n* = 93) of participants used smartphones for longer than 4 h per day, and 27.1% (*n* = 51) used their smartphone for more than 5 h per day.

Table 3 shows the relationship between smartphone use per day, lifestyle, social support, depression, and sleeping disorders. The Cronbach’s alpha was 0.78 for this part of the study. Overall, a relationship was found between the use of smartphones and female gender (*p* < 0.05), depression and longer smartphone use (*p* = 0.025), which is significant at the level of *p* < 0.05. In addition, bedtime (*p* = 0.000), longer sleeping hours (*p* < 0.001), and eating breakfast show a positive correlation with hours of smartphone use. However, surprisingly, this study did not find any significant correlation between sleeping disorders and hours of smartphone use.

Table 4 demonstrates the correlations between depression, sleeping disorders, hours of smartphone use, email, online chats, games, internet browsing, and TV. With this table, this paper not only tried to show the association between excessive hours of smartphone use and insomnia and depression, but also the relationship between the excessive use of smartphones for the specific variables of SNS, internet browsing, game, email, online chats, and online TV. 

The correlation table illustrates that there is a significant correlation between smartphone use and depression at (*p* < 0.05). Moreover, it was found that smartphone use has a strong significant relationship with SNS (*p* < 0.01), online chats (*p* < 0.01), internet browsing (*p* < 0.01), as well as online gaming (*p* < 0.01). Depression and insomnia also showed a strong correlation (*p* < 0.01). Furthermore, it was found that depression has a strong correlation with online chat services at the level of *p* < 0.01, and with SNS and internet browsing at the level of *p* < 0.05.

## 4. Discussion

The purpose of this study was to examine the relationship between the hours of smartphone use and depression and sleeping disorders among Korean college students. The results of this study show that the excessive use of smartphones by Korean college students has a close association with depression. Table 1 suggests that around 50% of the collected samples spent more than 4 to 5 h on their smartphones per day. This is higher than many other developed countries, such as Australia [74]. The current study provides evidence that excessive smartphone use is associated with depression. Students who use their smartphone for more than 4 to 5 h each day have a tendency to suffer from depression. However, sleeping disorders do not show a strong correlation with excessive smartphone use. The research done by Chokshi et al. [75] on mobile phone addiction with anxiety, depression, stress, and sleep quality among college students of Surat, India has also not found a strong correlation between the excessive use of mobile phone and sleep quality. However, a moderate relationship was found. Another research done by Musetti et al. [76] found a contradictory result between mobile phone addiction and playing video games. In particular, this research found a negative correlation between mobile gaming and mobile phone use among the female population in Italy. The majority of the earlier research has found that there is a close association between smartphone addiction and sleeping disorder. However, this present study did not find such a correlation, due to several explainable reasons. Firstly, if we look into the demographic data of the collected samples from Table 1, we can see that the majority of the students considered for this research were senior students (40.4%). In South Korea, senior students concentrate on their academic career since they need to find jobs right after they graduate. Therefore, they spend less time on playing online games and use their smartphones mostly for other purposes. In this COVID-19 situation, most of the lectures are taken via Zoom or online YouTube video materials. In addition, students are taking long hours of online classes, which may be the reason the data show excessive use of smartphones, but not due to smartphone addictions. Therefore, there is a possibility of not having a correlation between the excessive use of smartphones and sleeping disorders. Furthermore, data from Table 2 also indicate that around 50% of the collected samples are not playing mobile games at all, which might be another reason for not having a correlation between the excessive use of smartphones and sleeping disorders. In general, students play online games late at night and get addicted, which cause sleeping disorders [24,77,78]. On the other hand, excessive smartphone use (4 to 5 h or more) for social networking sites (*p* < 0.014); online chats (*p* < 0.006); and internet browsing (*p* < 0.019) have a significant relationship with depression (Table 4). Previous studies performed in Hong Kong show similar results. Participants in that study also showed a relationship between excessive mobile phone use and poor sleep quality. In addition, another study performed on Japanese high school students stated that excessive use of mobile phones has a significant correlation with less sleep and weakness [24]. The results of this study suggested that there is a significant correlation between sleeping disorders and depression (*p* < 0.000). 

The present paper also has some limitations. Firstly, we should note that this research paper used a self-reported questionnaire to understand the amount of time students spent on their smartphones. However, several studies explained that an individual might not be certain of the amount of time they spend on their smartphone [79,80]. The sample size of the present study was comparatively small and was confined to two universities in South Korea. However, both universities have fairly equally distributed students from all parts of South Korea, with a student capacity of 24,000 and 16,000. Therefore, the sample fairly represents the whole student population. However, collecting samples from several other universities will give a better perspective in terms of the entire student population. Lastly, this study does not ask any kinds of personality questions (the Five Big Personality Traits). Students that are introverted by nature and tend to spend more time at home may use smartphones more and may not experience any kind of depression. Future studies should consider personality traits in order to further explore the subject. Furthermore, this research can be extended by thoroughly adding up the impact of COVID-19 and smartphone use and depression among university students in South Korea. Researchers can also compare how foreign students cope with smartphone use and depression than Korean university students. Finally, a new complementary qualitative study can be done after collecting nation-wide students’ data to explore the relationship between the use of smartphones, more specifically social media and evidence of psychological problems and sleep disorders. Even though this paper has several limitations, meaningful insights can be gained. The current study helps us understand that excessive smartphone use has an association with depression. Therefore, a balanced use of smartphones is essential to a healthy, happy life.

## 5. Conclusions

The results of the current study provide evidence that the overuse of smartphones is linked with depression among university students. This research also expanded the literature to include the excessive use of smartphones, depression, and sleeping disorders in university students. Moreover, depression is strongly correlated with sleeping disorders. Furthermore, this study found a close relationship between the excessive use of SNS, online chats, and internet browsing via smartphones and depression. A balanced use of smartphones should be adopted, and university faculties should educate students on how to limit smartphone use and protect themselves from smartphone addiction. 

## Figures and Tables

**Table 1:** Socio-demographic variable.

Parameters	Mean	SD
Athens Insomnia Scale (range 0 to 24)	7.1	3.8
CES-D (range 0 to 33)	8.4	5.4
Parameters	*n*	%
School Year		
Freshmen	24	14.3
Sophomore	47	25
Junior	38	20.2
Senior	76	40.4
Gender		
Male	95	50.5
Female	93	49.46
Poses Smartphone		
Smartphone	187	99.5
2G Phone	1	0.5

**Table 2:** Time of mobile-tool use.

		None	<1 h	1–2 h	2–3 h	3–4 h	4–5 h	>5 years
Smartphone Use	Number	0	2	19	27	47	42	51
	**%**	0	1	10	14.3	25	22.3	27.1
		None	<30 min		30 min–1 h	1–2 h		>2 h
E-mail	Number	56	112		12	7		1
	%	29.7	59.5		6.3	3.7		0.5
Online Chatting	Number	29	23		48	-		-
	%	15.4	12.2		25.5	0		0
Social Media	Number	25	27		42	57		37
	%	13.2	14.3		22.3	30.3		19.6
Internet Browsing	Number	4	51		67	40		26
	%	2.1	27.1		35.6	21.2		13.8
Playing Games	Number	93	39		30	14		12
	%	49.4	20.7		15.9	7.4		6.3
Watching TV	Number	33	45		49	50		11
	%	17.5	23.9		26	26.5		5.8
			No problem		Problem			
Sleeping Disorders			36.1%		63.8%			
Depression			47.8%		52.1%			

**Table 3:** The relationship between the number of hours of smartphone use per day, lifestyle social support, depression, and sleeping disorders. *p*-Values were conducted by the Kruskal-Wallis test and Mantel-Haenszel test.

Variables		Hours of Smartphone Use				Total	*p*-Value
		<1	1 to <3 h	3 to <5 h	≥5		
Sleeping disorders	No Problem(%)	50.0	39.1	37.1	31.4	36.2	<0.378
	Problem(%)	50.0	60.9	62.9	68.6	63.8	
Depression	No Problem(%)	100.0	60.9	43.8	41.2	47.9	<0.025
	Problem(%)	0.0	39.1	56.2	58.8	52.1	
What is your gender?	Male(%)	100.0	65.2	43.8	47.1	50.5	<0.041
	Female(%)	0.0	34.8	56.2	52.9	49.5	
Participating in club activities	Sport(%)	50.0	19.6	19.1	19.6	19.7	<0.968
	Culture and Others(%)	0.0	26.1	30.3	29.4	28.7	
	None(%)	50.0	54.3	50.6	51.0	51.6	
When do you go to bed?	Before 11 p.m.(%)	0.0	8.7	7.9	0.0	5.9	<0.000
	Between 11 and Midnight(%)	0.0	26.1	11.2	11.8	14.9	
	Between 12 and 1 a.m.(%)	100	45.7	47.2	33.3	43.6	
	After 1 a.m.(%)	0.0	19.6	33.7	54.9	35.6	
When do you wake up?	Before 6 a.m.(%)	0.0	6.5	0.0	3.9	2.7	<0.275
	Between 6 and 7 a.m.(%)	100	54.3	39.3	37.3	43.1	
	After 7 a.m.(%)	0.0	39.1	60.7	58.8	54.3	
How long do you sleep?	<5(%)	0.0	8.7	2.2	7.8	5.3	<0.000
	5 to <6 h(%)	50.0	39.1	39.3	35.3	38.3	
	6 to <7 h(%)	50.0	37.0	42.7	39.2	40.4	
	≥7 h(%)	0.0	15.2	15.7	17.6	16.0	
Do you eat breakfast?	Always(%)	50.0	41.3	14.6	17.6	22.3	<0.031
	Sometimes(%)	50.0	21.7	49.4	39.2	39.9	
	Do not eat(%)	0.0	37.0	36.0	43.1	37.8	
Do you communicate with your family?	Always(%)	50.0	56.5	69.7	66.7	65.4	<0.275
	Sometimes(%)	50.0	43.5	30.3	33.3	34.6	

**Table 4:** Pearson correlations between depression, insomnia, hours of smartphone use, and details of smartphone use using correlation analyses.

		Depression	Sleeping Disorder	Smartphone Use	Email	SNS	Online Chatting	Internet Browsing	Game	TV
Depression	Correlation	1	0.431 **	0.164 *	0.059	0.179 *	0.201 **	0.171 *	0.125	0.138
	Sig.		0.000	0.025	0.420	0.014	0.006	0.019	0.088	0.059
Sleeping Disorder	Correlation		1	0.065	0.004	0.030	0.066	0.073	0.075	0.055
	Sig.			0.378	0.962	0.681	0.365	0.316	0.307	0.452
Smartphone Use	Correlation			1	0.060	0.421^**^	0.375^**^	0.333^**^	0.213	0.542
	Sig.				0.416	0.000	0.000	0.000	0.073	0.081
Email	Correlation				1	0.161	0.183	0.179	0.071	0.137
	Sig.					0.088	0.072	0.064	0.331	0.060
SNS	Correlation					1	0.225	0.018	0.201	0.261
	Sig.						0.070	0.809	0.069	0.059
Online chatting	Correlation						1	0.155	0.154	0.067
	Sig.							0.063	0.054	0.359
Internet Browsing	Correlation							1	0.238	0.094
	Sig.								0.081	0.199
Gaming	Correlation								1	0.157
	Sig.									0.057
TV	Correlation									1
	Sig.									

** Correlation is significant at the 0.01 level (two-tailed); * correlation is significant at the 0.05 level (two-tailed).

## Data Availability

Not applicable.

## Appendix A

**Table A1:** Original item questions of the Center for Epidemiologic Studies Depression Scales (CES-D).

		No Problem (1)	Slightly Delayed (2)	Markedly Delayed (3)	Very Delayed or Did Not Sleep at All (4)
1	Sleep induction (time takes to fall asleep after turning off the lights).				
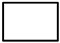
2	Awakening during the night.				
3	Final awakening earlier than desired.				
4	Total sleep duration.				
5	Overall quality of sleep (no matter how long you slept).				
6	Sense of well-being during the day.				
7	Functioning (physical and mental) during the day.				
8	Sleepiness during the day.				

## Appendix B. Athens Insomnia Scale (AIS)

**Table A2:** This scale is intended to record your own assessment of any sleep difficulty you might have experienced. Please, check (by circling the appropriate box) the items below to indicate your estimate of any difficulty, provided that it occurred at least three times per week during the last month.

		During the Past Week			
		Rarely or None of the Time (<1 Day)	Some or a Little of the Time (1–2 Days)	Occasionally or a Moderate Amount of Time (3–4 Days)	Most or All the Time (5–7 Days)
1	I was bothered by things that usually do not bother me.				
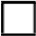
2	I did not feel like eating; I had no appetite.				
3	I felt that I could not shake off the blues even with help from my family or friends.				
4	I felt that I was just as good as other people.				
5	I had trouble keeping my mind on what I was doing.				
6	I felt depressed.				
7	I felt that everything I did was an effort.				
8	I felt hopeful about the future.				
9	I thought my life had been a failure.				
10	I felt fearful.				
11	My sleep was restless.				
12	I was happy.				
13	I talked less than usual				
14	I felt lonely.				
15	People were unfriendly.				
16	I enjoyed life.				
17	I had crying spells.				
18	I felt sad.				
19	I felt that people dislike me.				
20	I could not get “going”.				
